# Gender and healthy eating attitude strongly predict sustainable food literacy among Turkish young adults, while Mediterranean diet adherence shows only weak correlation

**DOI:** 10.3389/fpubh.2025.1606495

**Published:** 2025-07-09

**Authors:** Gizem Helvacı, Fatma Tayhan, Ümüş Özbey Yücel

**Affiliations:** ^1^Faculty of Health Sciences, Department of Nutrition and Dietetics, Mehmet Akif Ersoy University, Burdur, Türkiye; ^2^Faculty of Health Sciences, Department of Nutrition and Dietetics, Cankiri Karatekin University, Çankırı, Türkiye; ^3^Faculty of Health Sciences, Department of Nutrition and Dietetics, Amasya University, Amasya, Türkiye

**Keywords:** sustainability, food literacy, Mediterranean diet, nutrition attitudes, healthy lifestyle

## Abstract

**Background:**

Sustainability is the main concept shaping Turkey’s development plans. Adherence to healthy and sustainable diets can significantly contribute to development goals. Based on the sustainability agenda, we aimed to determine the level of sustainable food literacy among adults. We also evaluated the effect of Mediterranean diet adherence and healthy eating attitude on sustainable food literacy.

**Methods:**

A survey form containing scales for sustainable food literacy (SFLS), adherence to the Mediterranean diet (MEDAS), and attitudes toward healthy eating (ASHN) was administered to 319 Turkish adults aged 19–40.

**Results:**

40% of individuals have a low SFLS score, while 55% have a low MEDAS score. Gender affects SFLS scores, with men scoring lower (β = −0.31, *p* < 0.001). ASHN scores positively affect SFLS scores (β = 0.51, *p* < 0.001). Individuals’ SFLS scores are weakly positively correlated with MEDAS scores (r = 0.14, *p* = 0.013).

**Conclusion:**

Female gender and positive nutritional attitudes are major predictors of sustainable food literacy. Educational programs should be instituted to redefine gender roles, promote male engagement in environmental sustainability through the equitable distribution of domestic tasks, and positively influence individuals’ attitudes toward healthy eating. The observation that persons possessing high sustainable food literacy do not completely conform to the Mediterranean diet underscores the necessity to identify and address the issues beyond knowledge that hinder adherence to this dietary regimen through policy interventions.

## Introduction

The spread of obesity and climate change has required a global transformation in diets and food systems towards sustainable practices (1). Diets and activities in food supply chains are estimated to be responsible for about one-third of human-induced climate change (2). In reducing the environmental impact of diet, the Mediterranean diet, which involves reducing the quantity of animal-based products, changing their types, and is culturally acceptable and nutritionally adequate, is considered appropriate. However, with the change in lifestyles and consumer preferences towards processed convenience foods, even countries in the Mediterranean region have gradually moved away from this dietary model (3). Nutrition is an area where everyone can contribute to climate change. Therefore, both environmental awareness and food literacy are important in combating climate change (4). Food literacy is an important determinant in maintaining the diet quality of individuals and communities (5). The relationship between food literacy and healthy eating has also included environmental sustainability, leading to the emergence of the term ‘sustainable food literacy.’ Sustainable food literacy includes the knowledge, skills, attitudes, and practices necessary for adapting to sustainable diets (6). The sustainable food literacy scale was developed in 2022 and has been used in limited research. This study focuses on determining the level of sustainable food literacy of adults and asserts that individuals with superior nutritional attitudes and behaviors may exhibit greater sustainable food literacy.

Sustainable development is a global goal aimed at balancing human well-being with ecological well-being. The sustainable development goals set by the World Health Organization (WHO) include issues such as poverty, hunger, access to clean water, gender equality, economic growth, combating climate change, and conscious production and consumption (7). The adoption of healthy eating models is important for ensuring sustainable development as a way to prevent food insecurity and malnutrition (8). Many features of the Mediterranean diet are compatible with sustainable development goals. The mentioned features are as follows: (1) reduces dependence on livestock; (2) supports local farmers; (3) promotes sustainable agriculture; (4) preserves biodiversity; (5) prevents diseases with high fiber, polyphenols, and nutrient content. It has been suggested to prioritize the implementation of the Mediterranean diet globally to achieve sustainable development goals (7). Our study will provide up-to-date information on the adherence of young adults to the Mediterranean diet. Information about young generations’ adherence to the Mediterranean diet is important because it reflects trends towards a sustainable future.

Healthy eating attitude is a multifaceted concept that encompasses beliefs, preferences, and behaviors towards food. It has been reported that the education provided to develop sustainable nutrition literacy positively affects individuals’ attitudes towards healthy eating (9). The positive development of attitudes towards nutrition can facilitate the easier adoption of dietary practices that can reduce health risks (10). Previously, the triadic relationship between young adults’ sustainable food literacy, adherence to the Mediterranean diet, and attitudes towards healthy eating had not been investigated. We aimed to fill the gap related to the topic. We believe that the results obtained from this study will be useful in planning effective strategies to change the dietary behavior of the community towards recommendations.

## Materials and methods

319 young adults participated in the cross-sectional study conducted in Turkey between July and October 2024. Those who volunteered to participate in the study and were between the ages of 19 and 40 without communication problems were included. The study eliminated participants who had illnesses or situations that could interfere with normal diet, such as pregnancy or lactation. Using correlation analysis and the G-Power analysis tool, the sample size was determined. The effect size was calculated using data from a study that looked at the connection between adult Mediterranean diet adherence scores and food literacy (11). We set out to reach at least 171 participants with an effect size of 0.245, a Type 1 error (*α*) of 0.05, and 95% power. In the end, 319 young adults (69% female, 31% male) participated in the study.

The data were collected through an online survey link sent to the participants. Through well-known people, the poll was distributed to groups formed on social media sites. The permission form portion on the first page of the questionnaire explained the goal of the study and the participants’ freedom to discontinue participation at any moment. A questionnaire comprising general information, dietary habits, a sustainable food literacy scale, a healthy eating attitude scale, and a Mediterranean diet adherence scale was completed by study participants who consented to participate. The Helsinki Declaration’s guidelines were followed when conducting the study.

### Sustainable food literacy scale (SFLS)

The scale is used to evaluate adults’ (consumers, food service workers, students, etc.) level of sustainable food literacy and related abilities. Developed by Teng and Chih in 2022, the Turkish validity and reliability study was conducted by Kubilay and Yüksel in 2023 (6, 12). It consists of a total of 26 items on a 7-point Likert scale. While the original scale consists of four sub-dimensions, five sub-dimensions have been defined for Turkey (sustainable food knowledge I, sustainable food knowledge II, cooking and kitchen skills, attitudes, intention to act, and strategies). The scale yields a total score between 26 and 182, where high values indicate a high level of sustainable food literacy (12). As a result of the Receiver Operating Characteristics (ROC) analysis, the cutoff point for the Sustainable Food Literacy Scale was determined to be 130.5. Accordingly, those scoring below 130.5 are classified as having low sustainable food literacy, while those scoring 130.5 and above are classified as having high sustainable food literacy.

### Attitude scale for healthy nutrition (ASHN)

The scale includes items on food content and balanced eating habits and shows how important a healthy diet is for the individual. Demir and Cicioğlu (13) developed the scale and conducted a Turkish validity and reliability study. It is a 21-item, 5-point Likert-type scale with 4 sub-dimensions (knowledge about nutrition, feelings towards nutrition, positive nutrition, poor nutrition). The total score that can be obtained from the scale varies between 21 and 105, and increasing scores reflect a more ideal attitude (13).

### Mediterranean diet adherence scale (MEDAS)

The scale offers a quick evaluation of Mediterranean diet compliance. It was developed by Schröder et al. (2011) and the Turkish validity and reliability study was conducted by Pehlivanoğlu et al. (2020) (14, 15). The 14-item in scale inquiries about the primary oil used in meals, the quantity of olive oil taken daily, the type of meat that is most liked, the portions of fruits and vegetables, and the consumption amounts of certain foods (red meat, wine, legumes, nuts, fish, and shellfish, margarine-butter). Each item is scored as 1 or 0 according to the amount of consumption. Acceptable adherence to the Mediterranean diet is indicated by a total score of seven or higher, and strict adherence to the diet is indicated by a score of nine or higher (15).

### Statistical analysis

The IBM SPSS (Statistical Package for Social Sciences) 25.0 software program was utilized to evaluate the data. The mean, standard deviation, number, and percentage are the ways that descriptive statistics are displayed. The Student’s t-test was used to compare continuous variables, while the chi-squared test was used to evaluate categorical variables. Correlation analysis has been used to ascertain the correlations between the scale scores. The factors influencing the scores on the Sustainable Food Literacy Scale were identified using multiple linear regression analysis. In the analysis, SFLS scores were used as the dependent variable; independent variables such as gender, BMI, MEDAS, and ASHN were included in the model. A model adjusted for age has also been presented. In the evaluation of scores obtained from the Sustainable Food Literacy Scale, the cutoff point determined by ROC analysis was used. In the presence of significant threshold values, the sensitivity, specificity, positive predictive value, and negative predictive value of these thresholds were calculated. Cases where the type 1 error rate was less than 5% were considered statistically significant in terms of the test’s diagnostic value when analyzing the area under the curve (16).

## Results

Table 1 presents the distribution of general characteristics and scale scores according to gender. The rate of cigarette smokers was higher among men (39.0%) than women (19.2%) (*p* < 0.001). Alcohol use was also more common among men (32.0%; *p* = 0.001). Eating speed differed significantly according to gender (<0.001). Among men, 13.0% reported eating slowly, 47.0% reported eating moderately, and 40.0% reported eating fast. In women, 16.9% reported eating slowly, 70.8% reported eating moderately, and 12.3% reported eating fast. The habit of exercising regularly was more common among men (*p* < 0.001). The mean BMI of the study participants was 24.5 ± 3.5 kg/m^2^ in men and 22.5 ± 4.0 kg/m^2^ in women (*p* < 0.001). The mean ASHN, MEDAS, and SFLS scores of men were statistically significantly lower than those of women (*p* < 0.05). In addition, the mean SFLS sub-dimension scores (sustainable food knowledge I, sustainable food knowledge II, food and culinary skills, attitudes, intention to take action, and strategies) were lower in men (*p* < 0.001).

**Table 1:** Comparison of participants’ general characteristics and scale scores by gender.

Variables	Gender				Total (*n* = 319)		*p*^a^
	Male (*n* = 100)		Female (*n* = 219)				
	*n*	%	*n*	%	*n*	%	
Smoking habit							
Yes	39	39.0	42	19.2	81	25.4	<0.001**
No	61	61.0	177	80.8	238	74.6	
Alcohol Use							
Yes	32	32.0	34	15.5	66	20.7	0.001*^b^
No	68	68.0	185	84.5	253	79.3	
Skipping main meals							
Yes	38	38.0	97	44.3	135	42.3	0.291
No	62	62.0	122	55.7	184	57.7	
Eating speed							
Low (Slow)	13	13.0	37	16.9	50	15.7	<0.001**
Moderate (Medium)	47	47.0	155	70.8	202	63.3	
High (Fast)	40	40.0	27	12.3	67	21.0	
Perception of eating adequately and balanced							
Yes	45	45.0	91	41.6	136	42.6	0.564
No	55	55.0	128	58.4	183	57.4	
Exercise regularly							
Yes	44	44.0	50	22.8	94	29.5	<0.001**
No	56	56.0	169	77.2	225	70.5	
	**X ± SD**		**X ± SD**		**X ± SD**		***p* ^c^**
Age (year)	26.0 ± 9.4		22.9 ± 5.5		23.8 ± 7.1		<0.001**
BMI (kg/m^2^)	24.5 ± 3.5		22.5 ± 4.0		23.1 ± 3.9		<0.001**
ASHN total score	68.4 ± 9.2		72.3 ± 11.5		71.0 ± 10.9		0.003*
MEDAS total score	6.0 ± 1.8		6.5 ± 1.8		6.3 ± 1.8		0.040*
SFLS total score	104.3 ± 43.9		139.5 ± 35.6		128.4 ± 41.7		<0.001**
SFLS subscriptions							
S1	23.1 ± 10.7		31.0 ± 9.4		28.5 ± 10.5		<0.001**
S2	16.9 ± 7.8		22.0 ± 6.2		20.4 ± 7.2		<0.001**
S3	24.7 ± 11.5		32.7 ± 9.2		30.2 ± 10.6		<0.001**
S4	11.7 ± 6.2		15.6 ± 4.9		14.4 ± 5.6		<0.001**
S5	27.8 ± 12.7		37.9 ± 10.2		34.7 ± 12.0		<0.001**

*p < 0.05, **p < 0.001, BMI, Body Mass Index; ASHN, Attitude Scale for Healthy Nutrition; MEDAS, Mediterranean Diet Adherence Screener; SFLS, Sustainable Food Literacy Scale; S1, Sustainable Food Knowledge 1; S2, Sustainable Food Knowledge 2; S3, Cooking and Kitchen Skills; S4, Attitudes; S5, Intention to take Action and Strategies; X ± SD, mean ± deviation.

^a^Pearson’s chi-squared was used.

^b^Continuity correction test was used.

^c^Student t test was used.

In Table 2, some characteristics of individuals have been compared according to their level of sustainable food literacy. Those with high sustainable food literacy have a lower average age (*p* < 0.001). While 51.9% of men have low sustainable food literacy, the vast majority of women (82.6%) have high sustainable food literacy (*p* < 0.001). In the group with low sustainable food literacy, the rates of smoking and alcohol consumption are higher (*p* < 0.001). The average number of meals and meal skipping rates are similar among individuals with high and low sustainable food literacy (*p* > 0.05). Those with high sustainable food literacy believe they eat a sufficient and balanced diet at a higher rate (48.4%; *p* = 0.011). The rate of those who exercise regularly was 38.0% in the group with low sustainable food literacy and 23.7% in the group with high sustainable food literacy, and the difference between the groups was significant (*p* = 0.006). Individuals in the group with low sustainable food literacy had higher BMI scores and lower ASHN and MEDAS average scores (*p* < 0.05).

**Table 2:** Comparison of some characteristics of individuals according to their sustainable food literacy level.

Variables	Sustainable food literacy				*p*
	Low *n* = 129		High *n* = 190		
		95% CI Lower-Upper		95% CI Lower-Upper	
Age^b^	25.9 ± 9.0	24.3–27.5	22.5 ± 5.0	21.7–23.2	<0.001**
Gender^a^					
Male	67 (51.9)	43.0–60.8	33 (17.4)	12.3–23.5	<0.001**
Female	62 (48.1)	39.2–57.0	157 (82.6)	76.5–87.7	
Smoking habit^a^					
Yes	47 (36.4)	28.1–45.4	34 (17.9)	12.7–24.1	<0.001**
No	82 (63.6)	54.6–71.9	156 (82.1)	75.9–87.3	
Alcohol Use^a^					
Yes	38 (29.5)	21.8–38.1	28 (14.7)	10.0–20.6	<0.001**
No	91 (70.5)	61.9–78.2	162 (85.3)	79.4–90.0	
Number of main meals^b^	2.41 ± 0.5	2.31–2.51	2.42 ± 0.5	2.34–2.50	0.939
Number of snacks^b^	1.57 ± 0.6	1.45–1.68	1.63 ± 0.6	1.53–1.72	0.426
Skipping main meals^a^					
Yes	53 (41.1)	32.5–50.1	82 (43.2)	36.0–50.5	0.713
No	76 (58.9)	49.9–67.5	108 (56.8)	49.5–64.0	
Eating Speed^a^					
Low (Slow)	25 (19.4)	13.0–27.3	25 (13.2)	8.7–18.8	0.022*
Moderate (Medium)	70 (54.3)	45.3–63.1	132 (65.9)	62.4–75.9	
High (Fast)	34 (26.4)	19.0–34.8	33 (17.4)	12.3–23.5	
Perception of eating adequately and balanced ^a^					
Yes	44 (34.1)	26.0–43.0	92 (48.4)	41.1–55.8	0.011*
No	85 (65.9)	57.0–74.0	98 (51.6)	44.2–58.9	
Exercise regularly^a^					
Yes	49 (38.0)	29.6–46.9	45 (23.7)	17.8–30.4	0.006*
No	80 (62.0)	53.1–70.4	145 (76.3)	69.6–82.2	
BMI (kg/m^2^)^b^	23.9 ± 4.1	23.1–24.6	22.6 ± 3.7	22.0–23.1	0.004*
ASHN total score^b^	64.7 ± 8.1	63.2–66.1	75.4 ± 10.5	73.9–76.9	<0.001**
IN subscale^b^	16.0 ± 6.0	14.9–17.0	21.9 ± 3.0	21.4–22.3	<0.001**
EN subscale^b^	17.9 ± 6.2	16.8–19.0	17.4 ± 4.5	16.8–18.1	0.422
PN subscale^b^	14.7 ± 5.1	13.8–15.6	18.2 ± 3.8	17.6–18.8	<0.001**
MN subscale^b^	15.9 ± 5.1	15.0–16.8	17.7 ± 4.9	17.0–18.4	0.002*
MEDAS total score^b^	6.0 ± 1.9	5.6–6.3	6.6 ± 1.7	6.3–6.8	0.004*

*p < 0.05, **p < 0.001, Numerical data are presented as mean ± standard deviation, and categorical data as frequency (*n*) and percentage (%). CI, Confidence Interval; BMI, Body Mass Index; ASHN, Attitude Scale for Healthy Nutrition; MEDAS, Mediterranean Diet Adherence Screener; IN, Information on Nutrition; EN, Emotion for Nutrition; PN, Positive Nutrition; MN, Malnutrition.

a Pearson’s chi-squared was used.

b Student t test was used.

**Figure 1:**
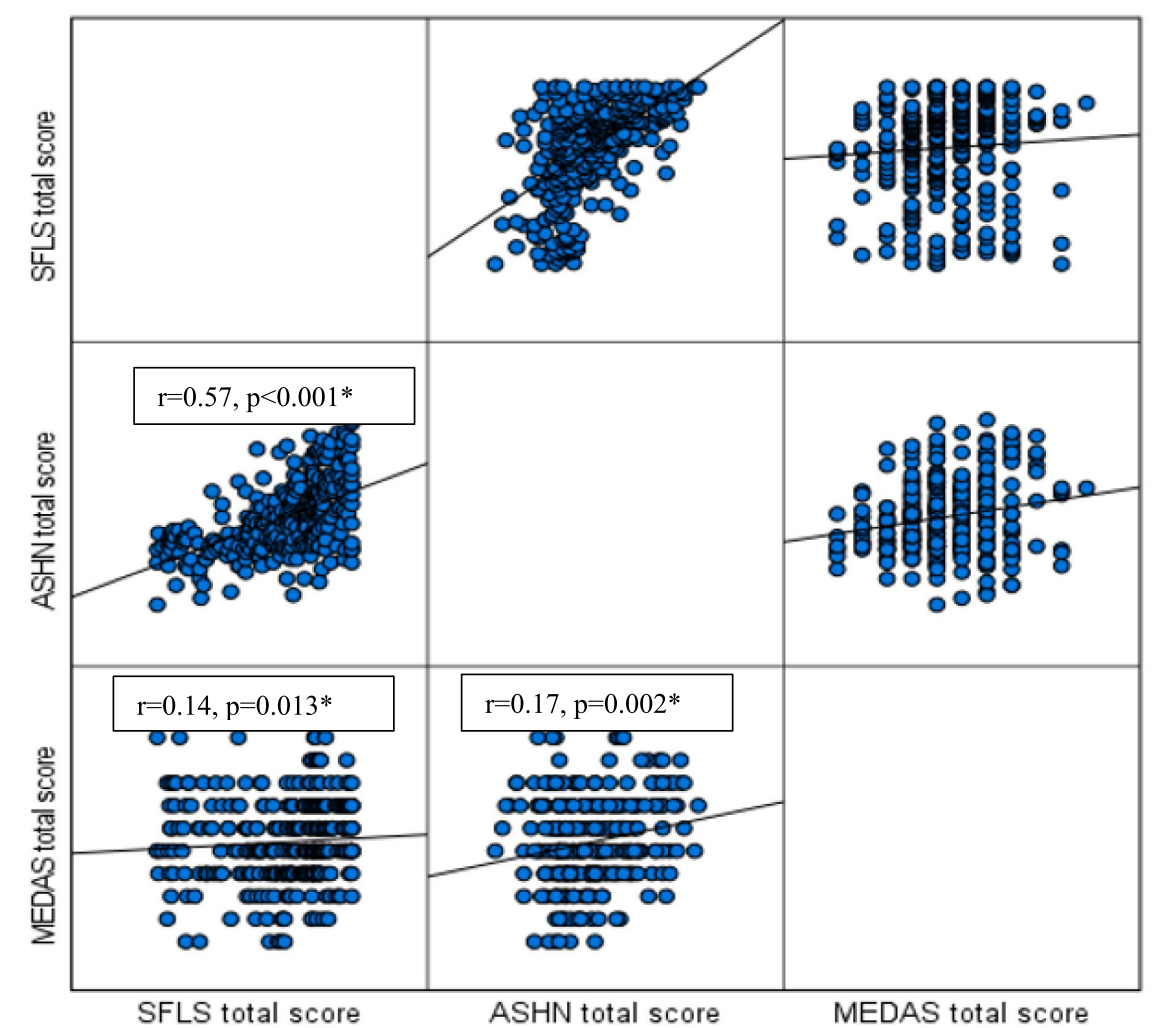
Correlation analysis between scales (each cell shows the distribution of the relationship between the two variables of interest). ASHN, Attitude Scale for Healthy Nutrition; MEDAS, Mediterranean Diet Adherence Screener; SFLS, Sustainable Food Literacy Scale.

The correlations between the scale scores are shown in Figure 1. There is a weak but significant positive relationship between individuals’ SFLS scores and MEDAS scores (r = 0.14, *p* = 0.013). Similarly, a weak positive correlation was found between ASHN scores and MEDAS scores (r = 0.17, *p* = 0.002). Additionally, SFLS and ASHN scores are moderately positively correlated (r = 0.57, *p* < 0.001).

Table 3 presents the results of multiple linear regression analysis. Gender had a significant effect on SFLS scores (β = −0.31, *p* < 0.001). The gender variable remained significant in the model adjusted for age (β = −0.29, *p* < 0.001). The negative β coefficient indicates that men have lower SFLS scores than women. BMI (β = −0.03, *p* = 0.44) and MEDAS scores had no significant effect on SFLS scores (β = −0.07, *p* = 0.10). In contrast, ASHN scores were found to have a significant and positive effect on SFLS scores (β = 0.51, *p* < 0.001). Significance was also maintained in the age-adjusted model (β = 0.51, *p* < 0.001) (Model 1). The positive β coefficient indicates that individuals with a more positive attitude towards healthy eating have higher SFLS scores.

**Table 3:** Modeling sustainable food literacy scale scores using multiple linear regression.

Variables	Unadjusted			Model 1		
	β	95%CI	*p*	β	95%CI	*p*
Gender (man or woman)	−0.31	−35.93; −20.20	<0.001*	−0.29	−33.80; −17.81	<0.001*
BMI	−0.04	−0.50; 0.20	0.40	−0.03	−0.48; 0.21	0.44
MEDAS	−0.07	−3.68; 0.33	0.10	−0.06	−3.41; 0.57	0.16
ASHN	0.51	1.61; 2.28	<0.001*	0.51	1.59; 2.26	<0.001*

*p < 0.001, CI: Confidence interval. BMI, Body Mass Index; ASHN, Attitude Scale for Healthy Nutrition; MEDAS, Mediterranean Diet Adherence Screener; Multiple linear regression analysis was used. Model 1: Adjusted for age.

## Discussion

One of the major results of our study is that gender and attitude towards healthy eating are strong determinants of sustainable food literacy. Men have a lower SFLS total score than women. Moreover, women had better scores in the sub-dimensions reflecting sustainable food knowledge, culinary skills, and the ability to translate their awareness of sustainability into action. Previous studies have also reported that women are more environmentally conscious and have a more ecological lifestyle. They were also found to be more willing to try new environmentally friendly and healthy foods (17, 18). Women have greater sensitivity, intuition, resilience, and compassion and are particularly sensitive to changes in nature (19). However, the observed gender-based disparities in our findings may be influenced more by societal gender roles than by inherent predispositions. As a consequence of the gender-based division of work, it is possible that women are taking on a greater share of the obligations that are associated with the kitchen and the home. Women devote much of their time and energy to their responsibilities at home. Their interest in sustainability has been attributed to their important role in the household economy and household food security (20). This circumstance puts women find themselves in the direct implementers of policies meant to increase sustainability. Therefore, many authors have emphasized the importance of integrating women’s perspectives and knowledge into natural resource management and empowering women to achieve sustainable development goals (19, 21).

In the current study, 60% of young adults have a high level of sustainable food literacy. Those with high sustainable food literacy have a lower average age. Sustainable lifestyles vary between generations. It has been reported that the younger generation is more environmentally conscious and supports sustainable development goals (22). Social media was used to obtain study data. Individuals that utilize social media platforms may be more exposed to sustainability content, causing specific views to emerge in the sample. It has been observed that social media significantly contributes to enhancing awareness and mobilizing action regarding environmental issues (23). It is important to take into account the potential that the findings may represent a particular social media user. Nonetheless, the cultivation of environmentally conscious behaviors extends beyond the impact of social media and is influenced by a variety of factors. These factors include participation in cultural activities, consumption practices within the family, responsibilities, and education (24–26). However, our research did not directly address these factors. In this study, the fact that those with high sustainable food literacy are younger indicates that age is an important factor in sustainable consumption. Previous studies have supported our findings by reporting that young people have higher levels of environmental awareness, concern, and attitudes (27, 28). Due to the utilization of comprehensive scales in our investigation, the participants had a substantial response burden. Because of this, despite the fact that we only focused on the age variable, we believe that the interpretative power should be expanded by exploring the effects of many factors in future studies.

Young adults’ attitude scores towards healthy eating positively affected their sustainable food literacy levels. There are different types of literacy used to improve health, including health, food, and nutrition literacy. Previous studies have reported that individuals with high food and nutrition literacy are more likely to adopt attitudes and behaviors related to healthy eating (29–31). By incorporating various aspects of sustainability into food and nutrition literacy, sustainable food literacy is expected to further promote healthy and sustainable living habits (6, 32, 33). However, literacy, knowledge, and education are closely related concepts, and individual attitudes depend on the interaction of these three (33). The literature has offered information on how attitudes are impacted by literacy level. This study makes the case that raising the degree of sustainable food literacy can be achieved by cultivating and positively altering eating attitudes.

The Mediterranean diet has become more recognized over time for its health benefits and cultural and environmental impacts. However, factors such as long preparation time of healthy foods, high cost of foods, accessibility, and culture may hinder adherence to the Mediterranean diet (34). In this study, most young adults (55%) adhered to a low Mediterranean diet. 33% of individuals had moderate adherence, and 12% had high adherence. Previous studies have reported moderate adherence of young adults to the Mediterranean diet (35, 36). Socioeconomic and cultural transformations may have contributed to our results. With the proliferation of single-person households, individuals are more likely to experience eating alone. This leads to both a lack of cooking skills and an over-reliance on takeout and processed foods in younger generations (37).

In the current study, adults with higher sustainable food literacy had higher Mediterranean diet adherence scores. SFLS and MEDAS scores showed a weak positive correlation. High food literacy, in other words, good nutritional knowledge and skills, has been identified as one of the factors facilitating adherence to the Mediterranean diet (34). Therefore, improving sustainable food literacy may be a good strategy to increase adherence to the Mediterranean diet. It may also positively influence other lifestyle variables. In this study, individuals with high sustainable food literacy had lower rates of smoking and alcohol consumption. Alcohol and cigarette consumption are unhealthy habits that contribute to greenhouse gas emissions and global warming (38, 39). It has been reported that individuals with these habits are less knowledgeable and less concerned about environmental problems (40). It has been reported that increasing knowledge and awareness about sustainability can motivate young adults to quit harmful habits (41).

Our study revealed the current status of sustainable food literacy, healthy eating attitudes, and Mediterranean diet quality in Turkish young adults and the relationship between them. However, this study, which did not include an educational intervention, did not provide an improvement in sustainable food literacy levels and did not evaluate behavioral changes. Sustainable food literacy education is an intervention with the ability to raise people’s levels of knowledge and alter their perspectives and actions (42). The data presented in the current study will form an important basis for future experimental design studies. However, sustainable nutrition is not only a behavior that depends on individual awareness. It is directly related to the accessibility of food systems, economic conditions, cultural norms, and social environment (43). It is important to make regulations that will facilitate conscious choices for sustainable nutrition in society. Clear labels can be used for plant-based, seasonal, and local foods in markets and restaurants. Menus in public institutions (schools, hospitals, etc.) could be diversified with Mediterranean-style and low-environmental-impact foods. Markets supporting local producers could be expanded (44). Our study revealed that individuals often have difficulty translating their knowledge about sustainability into actual behavior. The regulations mentioned could support the adoption of sustainability at a societal level by reducing environmental barriers to translating knowledge into behavior.

### Strengths and limitations

The strength of this study lies in determining the literacy level of young adults regarding the globally significant topic of ‘sustainable food’ and examining the related factors. The accuracy of the data has been thoroughly examined. The reliability of the participants’ responses to the questions in the study was checked using cross-check questions with similar meanings. These cross-check questions were placed in different parts of the survey form. Participants who provided incomplete or misleading answers to these questions were excluded from the study. However, the study has several limitations. First, due to the lack of comprehensive demographic information about participants’ education, it was not possible to determine whether the high number of individuals with high sustainable food literacy in this study was attributable to their educational level. Second, the level of sustainable food literacy, nutrition attitude, and adherence to the Mediterranean diet among the participants were determined through their subjective evaluations of their own lives. Self-reported skills may not always reflect actual practices. Nevertheless, the constraints of self-reported data collection were mitigated by utilizing measures that had previously established validity and reliability, as well as by ensuring that participation was optional within the study. Third, this is a cross-sectional study that cannot determine causality but reveals the relationships between variables. Fourth, since the study was conducted online, only individuals with technological devices and Wi-Fi access participated. The collection of data using a web-based method may have increased participation among individuals with internet access and computer literacy, thus limiting the creation of a larger and more homogeneous sample and potentially creating selection bias. However, this bias was partially mitigated as the study focused on young adults, whose members generally have significantly more access to online tools. Considering that internet access is 95.5% among households in Türkiye, the study did not create a strong socioeconomic distinction in terms of reaching participants (45). Furthermore, no discrimination was made based on social class when distributing the survey form. Finally, questions related to sensitive topics (nutrition and lifestyles) may have caused social desirability bias.

## Conclusion

Our survey revealed that 40% of young individuals possess low sustainable food literacy. Gender and attitudes toward healthful eating were identified as the determinants of sustainable food literacy. We need to enhance understanding of the significance of environmental sustainability among the youth of today. Policies designed to promote this understanding should not only enhance individual knowledge but also address the ramifications of gender disparities and the impact of socio-cultural frameworks. Gender inequalities should be reduced, and everyone should bear equal responsibility for saving the environment through their eating choices. It is important for men to engage actively in meal planning, shopping, meal preparation, and the efforts to minimize food waste within the household. Campaigns may be initiated to enhance men’s awareness of food responsibility, and educational curricula might be developed that emphasize gender equality. Efforts in this area will advance the attainment of sustainable development objectives and the eradication of gender-based inequities.

The level of sustainable food literacy is influenced by the attitudes of individuals toward healthy nutrition, as underscored in our research. Favorable attitudes towards healthy nutrition seem to establish a cognitive framework for making more judicious choices within environmental, social, and cultural contexts. These attitudes can be positively improved through effective nutrition advice and guidelines. Policies should teach critical thinking so people can assess the social and environmental impacts of their dietary choices. In this way, it may be possible to permanently alter people’s attitudes about healthy nutrition and increase sustainable food literacy.

Most young adults (55%) have a low adherence to the Mediterranean diet. The weak correlation observed in our study implies that individuals who possess a high level of sustainable food literacy do not necessarily completely adhere to the Mediterranean diet. Although a positive attitude toward healthy eating may be associated with higher sustainable food literacy and facilitate healthier dietary practices, this does not necessarily lead to greater adherence to specific diets such as the Mediterranean diet. Therefore, dietary behaviors are influenced by elements beyond the level of knowledge. It is clear that dietary changes are needed to progress towards sustainable development goals. Policies must identify and tackle the obstacles that make it difficult to adhere to the Mediterranean diet, such as financial limitations, availability of nutritious foods, and prevailing cultural practices.

## Data Availability

The raw data supporting the conclusions of this article will be made available by the authors, without undue reservation.
